# 肺磨玻璃结节的诊治策略

**DOI:** 10.3779/j.issn.1009-3419.2019.07.07

**Published:** 2019-07-20

**Authors:** 宝东 刘

**Affiliations:** 100053 北京，首都医科大学宣武医院胸外科 Department of Toracic Surgery, Xuanwu Hospital, Capital Medical University, Beijing 100053, China

**Keywords:** 肺结节, 磨玻璃结节, 影像, 病理, 手术, Pulmonary nodules, Ground-glass nodule, Imaging, Pathology, Surgery

## Abstract

近年来，随着高分辨率计算机断层扫描（high resolution computed tomography, HRCT）肺癌筛查项目的开展，越来越多的肺磨玻璃结节（ground-glass nodule, GGN）被发现。由于GGN的出现常常提示与肺癌相关，因此本文对其分类、影像、病理、随访和手术等临床关注的相关问题加以综述。

## Abstract (en)

Recent widespread use of high resolution computed tomography (HRCT) for the screening of lung cancer have led to an increase in the detection rate of very faint and smaller lesions known as ground-glass nodule (GGN). However, it had been proved that GGN was well associated with lung cancer in previous studies. Terefore, the classifcation, imaging characteristics, pathological type, follow-up, suggested managements and other clinical concerns of GGN were reviewed in this paper.

## 1 概念

肺磨玻璃结节（ground-glass nodule, GGN）是指在高分辨率计算机断层扫描（high resolution computed tomography, HRCT）上表现为其内支气管及血管纹理可显示的密度轻度增高影。其病因可见于肿瘤、感染、局部出血或间质纤维化等。

## 2 分类

2006年Suzuki^[1]^根据实性成分的比例将SPN分为6型。目前常根据GGN内部是否含有实性成分，分为纯磨玻璃结节（pure GGN, pGGN）和部分实性结节（part-solid lesion），而pGGN及部分实性GGN又称为亚实性（sub-solid）结节^[2-4]^。

## 3 影像学恶性征象

Henschke研究认为^[5]^：GGN恶性概率高于实性结节。实性结节恶性率仅7%，部分实性结节恶性率为63%（10/16），pGGN恶性率为18%（5/28）， > 20 mm的结节恶性率为80%。结节的位置（上叶）^[6]^、大小^[7-10]^和CT值^[11-13]^、形态（空泡、胸膜凹陷、支气管充气征）^[10, 14-16]^、血管形态改变^[14, 17]^、实性成分^[18, 19]^对鉴别良恶性有很重要的价值。

## 4 病理

1995年Noguchi^[20]^将肺腺癌分为6种类型。2011年，国际肺癌研究学会（International Association for Study of Lung Cancer, IASLC）、美国胸科学会（American Thoracic Society, ATS）和欧洲呼吸学会（European Respiratory Society, ERS）联合发布了肺腺癌多学科新分类方案^[21]^。将肺腺癌分为：浸润前病变、微浸润腺癌（minimally invasive adenocarcinoma, MIA）及浸润性腺癌（invasive adenocarcinoma, IA）。浸润前病变分为不典型腺瘤样增生（atypical adenomatous hyperplasia, AAH）和原位腺癌（adenocarcinoma *in situ*, AIS）。浸润性腺癌包括：贴壁为主型、腺泡为主型、乳头为主型、微乳头为主型和实体为主型等。Murakami等^[22]^分析了347例IA期非粘液肺腺癌完全切除后5年无病生存率：贴壁型为99.0%、腺泡型为82.4%、乳头型为80.8%、实体型为73.6%、微乳头型为33.3%。

## 5 影像-病理相关性

AAH影像表现为pGGN、直径 < 5 mm（少数10 mm-20 mm）、形态规则，术后5年生存率为100%^[23, 24]^。

AIS病理类型多非粘液性。影像表现为pGGN或部分实性结节，直径 > 5 mm且 < 30 mm，CT值较AAH略高，术后5年生存率为100%^[25]^。

MIA病理类型多非粘液性。影像表现为部分实性结节，实性成分≤5 mm，直径 > 10 mm且 < 30 mm，形态有分叶、支气管充气征、血管改变、胸膜凹陷。多项研究证实，MIA完全切除（肺叶切或亚肺叶切）后，5年生存率达100%，累计复发率为0%^[26]^。

浸润性腺癌的贴壁为主型影像表现为部分实性结节或实性结节，直径 > 10 mm，实性成分 > 5 mm，预后与实性成分成反比。浸润性腺癌分为腺泡状、乳头状、微乳头状、实性为主型，影像表现为实性结节，直径 > 10 mm，较贴壁为主型预后差。

pGGN为均匀磨砂玻璃样阴影，进展很慢。病理基础为肿瘤细胞呈贴壁样沿肺泡间隔生长，肺泡壁增厚，但肺泡腔未完全闭塞；病理类型对应于AIS或AAH及MIA，极少数可表现为浸润性腺癌。直径 < 5 mm时通常为AAH；直径介于5 mm-30 mm，或者pGGN直径 > 6.5 mm、边界完整或CT上出现血管形态改变时为AIS的可能性较大^[27, 28]^。Sim等^[29, 30]^对48例pGGN进行根治性切除：32例肺叶切除，16例亚肺叶切除（4例段切除、12例楔形切除）。结节直径为12（5-30）mm。随访39（23-77）个月无死亡和复发。

部分实性结节可表现为分叶、胸膜凹陷、支气管充气征和血管改变等。病理基础为实性部分主要由纤维化或塌陷的肺泡结构构成。 < 5 mm的实性成分以MIA多见^[19]^。若实性部分直径 > 5 mm，则提示浸润性腺癌的可能性较大^[16]^。

目前多数学者认为GGN依循顺序式多阶段发展：在影像学上，GGN是从pGGN进展为部分实性结节或实性结节；在病理学上，从AAH到AIS、MIA，直至IA ^[31, 32]^。

## 6 随访

### 6.1 Fleischner学会肺结节管理指南^[3]^

2013年Fleischner学会提出了磨玻璃结节的诊疗指南，2017年进行了更新。新指南将5 mm的临界值提高为6 mm，且延长了随访间隔。pGGN结节 < 6 mm时可不必常规随访；结节在≥6 mm时建议6个月-12个月确定稳定性，之后每2年复查直至5年。部分实性结节 < 6 mm时无需常规随访；结节在≥6 mm时建议6个月-12个月确定稳定性，且实性成分 < 6 mm时，每年复查直至5年。

### 6.2 美国国立综合癌症网络（National Comprehensive Cancer Network, NCCN）肺癌筛查指南^[33]^

pGGN结节 < 6 mm时无需常规随访；结节在≥6 mm时建议6个月-12个月确定稳定性，之后每2年复查直至5年。部分实性结节实性成分 < 6 mm时无需常规随访；实性成分≥6 mm时建议3个月-6个月确定稳定性，且实性成分 < 6 mm时建议每年复查直至5年；实性成分≥6 mm且高度怀疑肺癌时建议行活检或手术切除。

### 6.3 2013年的美国胸科医师学会（American College of Chest Physicians, ACCP）肺结节指南^[34]^

对于≤5 mm的pGGN不建议进一步评估（证据级别2C），对于 > 5 mm的pGGN建议每年随访，至少随访3年（证据级别2C）；对于≤8 mm的部分实性结节建议第3、12、24个月随访，随后每年随访（证据级别2C），对于 > 8 mm的部分实性结节建议每3个月重复CT评估，对持续存在的结节采用PET、非手术活检和/或手术切除进一步评价（证据级别2C）。

### 6.4 2016年肺结节亚洲共识^[35]^

≤5 mm的pGGN建议每年复查1次； > 5 mm的pGGN建议每年复查1次，连续3年复查后，如果结节无明显变化，仍建议每年复查CT。≤8 mm的部分实性结节建议第3、12、24个月随访，随后每年随访； > 8 mm的部分实性结节建议每3个月复查，并抗炎治疗，对持续存在的结节采用PET、非手术活检和/或手术切除进一步评价。

## 7 观察指标

随访观察指标包括GGN的大小、CT值、实性成分%（C/T, 0.25-0.5）、肿瘤消失率（tumor shadow disappearance rate, TDR）、倍增时间（doubling time, DT）和容积倍增时间（volume double time, VDT）。C/T > 50%或TDR < 50%时往往提示浸润性癌的可能性较大^[36, 37]^。不同病理学类型的VDT也不同：AAH（988±470）d、AIS（567±168）d、MIA（384±212）d^[15]^。

## 8 自然转归

多项研究结果均提示约20%的pGGN在随访过程中病灶变大或进展为部分实性结节；约40%的部分实性结节在随访中增大或实变区增大。Kakinuma等^[38, 39]^研究发现，439例直径≤5 mm的pGGN中，394例持续稳定，45例（10%）生长，仅4例（1%）为恶性（IA或MIA），该4例平均随访3.6年。

## 9 手术适应证

### 9.1 高危因素

中老年人（55岁-74岁）、既往恶性肿瘤病史、家族史、长期吸烟史（> 30年，或戒烟年限 < 15年）或特殊职业接触史（石棉）等情况。

### 9.2 影像学上恶性征象

毛刺征、分叶征、胸膜凹陷、部分实性；动态观察发现GGN增大2 mm、实性成分增加；贴近脏层胸膜的周围型GGN可局部切除。

### 9.3 患者对于GGN极度焦虑，无法缓解。

## 10 术前检查

### 10.1 PET/CT

pGGN病变标准化摄取值（standardizeduptakevalue, SUV）较低，PET/CT检查价值有限，一般不推荐。PET/CT检查主要用于实性或部分实性结节（实性成分 > 10 mm）^[40]^。

### 10.2 胸部CT增强扫描

pGGN病变原则上不需要做CT增强扫描；但部分实性结节、病灶与肺血管关系密切或者怀疑淋巴结转移者可行胸部CT增强扫描。

### 10.3 纤维支气管镜

pGGN病变，术前支气管镜检查阳性率低^[41]^。

### 10.4 经皮肺穿刺活检

Fleischner学会推荐实性成分≥5 mm者^[42-46]^。

### 10.5 分期检查

一般不必做骨扫描、头颅MRI检查和腹部超声等检查^[40]^。

## 11 术前辅助定位

### 11.1 术前定位技术

CT引导下经皮肺穿刺注射医用胶、亚甲蓝、吲哚菁绿等液体材料，或放置微弹簧圈、Hook-wire等金属材料辅助定位^[47-50]^；经电磁导航支气管镜或虚拟支气管镜导航（virtual bronchoscopy navigation, VBN）注入染料等定位^[51]^。

### 11.2 术中定位技术

术中B超定位、术中立体解剖定位。

## 12 胸腔镜手术

### 12.1 切口选择

GGN首选治疗方式是胸腔镜手术，包括单孔胸腔镜、单操作孔胸腔镜、三孔胸腔镜、剑突下胸腔镜。

### 12.2 切除范围选择

手术切除范围选择有赖于病灶大小、形态、CT值、部位、CEA水平、分期、患者年龄（> 70岁）、心肺功能、术者经验等。由于正在进行的大规模随机对照临床研究（如日本的JCOG0802、JCOG0804和美国的CALGB140503）还没有发表最后结果，目前尚不能支持亚肺叶切除能够像肺叶切除一样实现对早期肺癌的手术治疗。①楔形切除：周围型病灶≤2 cm、高龄、肺功能不良者，要求切缘 > 2 cm；②肺段切除：病灶位于优势段内、高龄、肺功能不良；③肺叶切除：病灶 > 2 cm、病灶位于段间、肺功能良、IA/MIA。

Kim等^[52]^比较了胸腔镜亚肺叶切除（73例）术后3个月、12个月的肺功能优于肺叶切除（227例）（*P* < 0.001）；进一步分析发现，仅在左上叶和左下叶切除时有统计学差异（*P* < 0.05）。

影像学上C/T比≤0.25是楔形切除的良好指征^[30, 53-55]^。Okami等^[56]^评估133例高龄（≥75岁，心肺功能差、既往肺叶切除等）非小细胞肺癌（non-smll cell lung cancer, NSCLC）患者叶切与亚肺叶切除的5年生存率无显著差异（73.4% *vs* 67.6%, *P*=0.92）。Deng等^[57]^回顾性分析了叶切（2, 336例）和段切（212例）的总生存（overall survival, OS）和无复发生存，肿瘤 < 2 cm无差异，2 cm-3 cm处于临界值（*P*=0.05）。Nomori^[58]^在一项前瞻性研究纳入了179例段切患者，肿瘤大小影响5年生存率。Dembitzer等^[59]^在平衡肿瘤大小之后，发现叶切和亚肺叶切在生存率上无差异。

Hattori等^[60, 61]^回顾性分析发现IA在薄层CT上有和没有GGO成分与OS无关，但两组5年生存率存在统计学差异。与实性成分相关。Tsutani等^[62]^纳入的239例GGO成分大于50%的肺腺癌患者，结节直径大小平均1.8（0.7-3）cm，不同切除范围（叶切、段切、楔形切）的3年肿瘤无复发率无统计学差异。纳入的327例实性成分 > 50%的肺腺癌患者，结节平均直径2.1（0.6-3）cm，实性成分平均直径1.7（0.5-3）cm，*Cox*回归发现3年肿瘤无复发率与切除范围无关^[63]^。

Altorki等^[64]^对诊断为NSCLC的≤3 cm实性结节比较了肺叶切除（294例）和亚肺叶切除（53例）的结果，患者临床资料平衡后，二者10年生存率无差异（86% *vs* 85%）。Scheel^[65]^根据术者经验，分为低经验组（≤5年）、中等经验组（5年-15年）、高级经验组（> 15年），发现低经验组更多选择肺叶切除；中等经验组的患者生存率更高；三组间不影响围术期结果。一项关于医生年手术量对手术选择影响的调查研究发现，随着医生年手术量增加，叶切的比例也相应增加，而楔形切和全肺切的比例减少^[66]^。Meta分析指出，胸腔镜切除I期NSCLC患者在生存率上，肺叶切除优于亚肺叶切除（HR=1.45），肺叶切除与肺段切除相当（HR=1.03），肺叶切除优于楔形切除（HR=4.19）^[67]^。Hwang等比较了平衡后的叶切和亚肺叶切（各94例）的手术时间和住院时间均无差异；术后并发症和死亡率也无差异；OS和无复生存无差异；亚肺叶切组FEV1损失低，但无统计学差异^[57, 68]^。通过对821例AIS和6, 137例MIA分析后发现，超过99.9%的AIS和MIA并未出现淋巴血管侵犯，且相关的多中心前瞻性随机临床研究证实：对于T1-2N0期NSCLC患者，系统性淋巴结清扫和系统性淋巴结采样在长期生存上并无优势。故对于早期肺腺癌患者不推荐进行系统性淋巴结清扫。

Sim等^[29]^对42例pGGN患者进行系统淋巴结清扫或采样，平均清扫23（7-53）枚。病理包括AIS和IA，淋巴结均阴性。Wolf等^[69]^研究发现，亚肺叶切除组的OS和无复发生存低于叶切组；但当进行了淋巴结清扫后，两组局部复发率、OS、无复发相当。段切与楔形切相比，有更宽的切缘（1.5 cm *vs* 0.8 cm）、更多的淋巴结采集个数（4个*vs* 1个）和淋巴结分期升级（9% *vs* 1%）。ACOSOG Z0030研究针对T1/T2、N0/N1（非肺门）周围型肺癌比较纵隔淋巴结采样和清扫，结果没有统计学差异^[70]^。所以淋巴结清扫可以提高分期的准确性、发现隐匿N2、降低复发、提高生存（存疑）；但是也增加了并发症、引流量和住院时间。

## 13 肿瘤热消融

Kodama等^[71]^回顾性评价了射频消融治疗33例患者的42个GGN优势（≥50%）肺腺癌的临床结果，平均随访42个月，局部进展率为14.3%（6/42），6例中的4例再次消融，除1例脑出血死亡以外，均存活，1年OS和肿瘤特异性生存率分别为100%和100%，3年OS和肿瘤特异性生存率分别为96.4%（95%CI: 77.5-99.5）和100%，5年OS和肿瘤特异性生存率分别为96.4%（95%CI: 77.5-99.5）和100%。Iguchi等^[72]^回顾性评价了射频消融治疗16例患者的17个表现为GGN为主（≥50%）肺癌的临床结果，中位肿瘤随访61.5个月，首次和二次技术效率1年分别为100%和100%，2年为93.3%和100%，3年为78.3%和92.3%；中位患者随访65.6个月，1例患者11.7个月因其他癌症复发而死亡，其余16例均存活，1年OS和肿瘤特异性生存率分别为93.3%和100%，5年分别为93.3%和100%。Yang等^[73]^回顾性分析了微波消融治疗肺部周围型GGO（腺癌）的初步结果，51例肺部GGO患者经微波消融治疗后3年的无局部复发生存率、肿瘤特异性生存率和总生存率分别为98%、100%和96%。

## 14 多原发GGN

### 14.1 诊断标准

诊断多原发肺癌（multiple primary lung cancer, MPLC）的标准有Martini和Melamed（1975）标准^[74]^、Antakli（1995）标准和ACCP（2013）标准^[75]^。多原发肺癌具有非浸润性、多中心起源（基因检测证实）的特点^[76]^。

### 14.2 手术原则

外科术式的选择原则是：（1）主病灶优先，兼顾次病灶；（2）同一肺叶双原发或多原发结节：同期手术多采用肺叶切除；（3）同侧不同肺叶单发病灶：若患者肺功能允许，可采取同期手术，一般较大病灶所在的肺叶行肺叶切除术，小病灶采取肺楔形切除；若两病灶较小，可采用不同肺叶的亚肺叶切除；（4）当病灶分别位于两侧肺叶时，选择分期切除的手术原则是：①先切除中心型、进展较快、病灶较大或伴有纵隔、肺门淋巴结转移的主病灶，后切除周围型、进展较慢、病灶较小或无淋巴结转移的次病灶；②先切除对预后影响较大的病灶：如病灶较大、密度较高、实性成分较大、恶性征象明显、分期较晚的病灶；③两次手术间隔时间太短不利于患者初次手术后的恢复，增加二次手术的风险；而间隔时间太长又会增加未切除侧病灶进展和转移的风险，一般两次手术的时间间隔应在6周-8周。选择同期切除遵循的手术原则是：①安全：先进行切除范围小的一侧，确保对侧手术安全；②不安全：先切除主病灶，二期对侧手术。（5）关于淋巴结清扫：无论采取何种手术方式，系统淋巴结清扫都是必要的，它对延长肿瘤局部控制时间、提高治愈率及完善诊断分期均具有重要意义^[77, 78]^。

Yao等^[79]^对29例同时性多原发肺癌（synchronous multiple primary lung cancer, SMPLC）进行了胸腔镜手术，与分期手术相比，术后并发症（*P*=0.703）、引流时间（*P*=0.485）无差异。Tanvetyanon系统综述纳入了6个研究467例SMPLC患者，预后差的因素有老年（HR=1.40）、男性（HR=1.64）、单侧肿瘤（HR=1.45）、N1/N2（HR=1.68/1.94）^[80, 81]^。Ishikawa等^[82]^对93例SMPLC（26例双侧）进行了手术，5年OS为87%；分析发现双侧、淋巴结转移、亚肺叶切除者预后差。Shimada^[83]^对67例SMPLC手术切除（39例完全切除），多因素分析发现，主病灶大小和实性成分与预后相关，次要或残留病灶及是否生长、新发不影响预后。Liu等^[84]^对122例SMPLC手术切除，5年生存率为40.5%，多因素分析发现不吸烟、病理类似、GGO成分、非全肺预后良好。

## 15 术后处理

多数GGN术后病理提示早期肺腺癌不需要化疗和放疗。只有极少数部分实性结节，如果病灶较大或者合并淋巴结转移才需要化疗。目前研究还没有证实分子靶向药物治疗对GGN患者有好处，除非因为肺功能低下而无法切除时，才建议患者做基因检测，以防将来复发时考虑靶向治疗。同期多原发GGN如因肺功能因素无法行根治性切除时，可考虑化疗。
